# A hedonic perspective on exercise intentions: cross-sectional associations with intensity preference, tolerance, and passion

**DOI:** 10.3389/fpsyg.2026.1891893

**Published:** 2026-07-27

**Authors:** Filipe Rodrigues, Pedro Duarte-Mendes, Rita Soares, Diogo S. Teixeira

**Affiliations:** 1 ESECS, Polytechnic University of Leiria, Leiria, Portugal; 2 Research Center in Sports Sciences, Health Sciences and Human Development (CIDESD), Vila Real, Portugal; 3 Sport Physical Activity and Health Research and Innovation Center (SPRINT), Castelo Branco, Portugal; 4 Department of Sports and Well-Being, Polytechnic Institute of Castelo Branco, Castelo Branco, Portugal; 5 Faculty of Physical Education and Sport, Lusófona University, Lisbon, Portugal; 6 Research Center in Sport, Physical Education, Exercise and Health (CIDEFES), Lisbon, Portugal

**Keywords:** exercise, harmonious passion, intensity preference, intensity tolerance, intention, obsessive passion

## Abstract

**Introduction:**

This cross-sectional study examined the associations of exercise intensity preference and tolerance with passion and the intention to continue exercising.

**Objective:**

A total of 284 fitness center members (140 females, 144 males; M_*age*_ = 29.84, SD = 9.27) were recruited. Participants were independently stratified into light-to-moderate and moderate-to-vigorous profiles based on intensity traits.

**Results:**

Descriptive results showed that harmonious passion (HP) was consistently higher than obsessive passion (OP) across all profiles, peaking in the moderate-to-vigorous preference group (*M* = 5.47). Linear regression models, explaining 8%–26% of the variance (*R*^2^ = 0.08–0.26), indicated that HP was significantly associated with stronger exercise intentions across all groups (β = 0.53 in the moderate-to-vigorous tolerance stratum). Conversely, OP displayed negative associations with intention, particularly in the moderate-to-vigorous tolerance group (β = –0.18). Furthermore, intensity preference was positively associated with intentions.

**Discussion:**

These findings suggest that individuals who self-select their training intensity may report higher persistence intentions due to positive hedonic responses. We conclude that exercise prescription may benefit from accounting for individual intensity preferences and fostering harmonious passion to support positive exercise intentions.

## 1 Introduction

According to the European Commission (2018), an estimated 68% of the Portuguese population is physically inactive. This high prevalence of physical inactivity can be attributed to two primary factors: the small number of individuals who opt to initiate a regular physical activity program and the alarmingly high dropout rates among those who do (Rodrigues et al., 2021). Although previous studies have consistently highlighted motivational and emotional factors as critical determinants of exercise behavior, attrition rates in fitness center settings remain exceptionally high (Rodrigues et al., 2018; Teixeira et al., 2012). Consequently, there is a pressing need to investigate novel theoretical frameworks and alternative factors that may be associated with long-term exercise adherence.

### 1.1 Hedonic Theory and affective responses

To elucidate the mechanisms underlying exercise adherence, the relationship between exercise and affective responses has become a primary focus in exercise psychology (Teixeira et al., 2021a). Rooted in Hedonic Theory, human behavior is driven by the pursuit of pleasure. From this perspective, an individual’s affective response to an activity dictates whether that behavior will be repeated (Kahneman, 1999). Specifically, the pleasure or displeasure experienced during physical exercise acts as a crucial determinant of long-term adherence, although it interacts with and competes against various other biopsychosocial and environmental factors (Ekkekakis and Dafermos, 2012). Affect is conceptualized as a neurophysiologically accessible state of pleasure or displeasure (Russell, 1980). It is the most basic building block of all emotional responses. According to the circumplex model of affect, these responses can be mapped across two orthogonal dimensions: valence (pleasure vs. displeasure) and perceived activation (high vs. low) as described in the literature (Barrett, 2006; Barrett and Russell, 1999). In recent years, authors have argued that the role of affect in exercise motivation has been underestimated in contemporary theoretical work (Wienke and Jekauc, 2016). Drawing from the Affect and Health Behavior Framework, positive affective responses, or positive shifts in hedonic valence, are linked to behavioral repetition, whereas negative affective responses prompt behavioral avoidance (Cabanac, 1992; Stevens et al., 2020; Williams and Evans, 2014). Therefore, when individuals experience positive feelings during exercise, the probability of repeating the activity significantly increases (Strobach et al., 2020).

### 1.2 Exercise intensity, preference, and tolerance

In the pursuit of understanding affective responses, exercise intensity emerges as a crucial variable. The relationship between exercise intensity and affect is conceptualized as an “inverted-U” dose-response curve. While moderate intensities, approaching 60%–70% of maximal aerobic capacity, tend to produce significant affective benefits, intensities that are excessively low or exceedingly high are ineffective at fostering positive affective changes (Ekkekakis et al., 2005b,^2008^). Specifically, exercising above the ventilatory threshold frequently triggers a decline in affective valence, resulting in displeasure and, subsequently, program dropout (Ekkekakis et al., 2008; Williams, 2008).

Because individual physiological and psychological differences heavily influence affective responses, adopting a rigid, “one-size-fits-all” approach to prescribing exercise intensity can be counterproductive, as uniform prescriptions fail to account for unique individual thresholds. To address the limitations of generalized intensity prescriptions, recent literature has shifted focus toward the preference for and tolerance of exercise intensity. Preference refers to an individual’s predisposition to select a specific intensity level, whereas tolerance is the trait influencing one’s capacity to continue exercising at a given intensity (Ekkekakis et al., 2005a; Teixeira et al., 2021a). Research on self-selected intensity demonstrates that individuals intuitively adjust their workout parameters to maintain a constant, positive rating of pleasure, typically selecting an intensity just below the aerobic-to-anaerobic transition (Cabanac, 1986; Lind et al., 2005). When prescribed exercise aligns with an individual’s intensity preference and tolerance, they experience higher levels of intra-exercise pleasure, creating the optimal conditions for the behavior to become a long-term habit (Teixeira et al., 2021a).

### 1.3 The Dualistic Model of Passion

This sustained pleasure and energetic dedication to a behavior over time is linked to the concept of passion. The Dualistic Model of Passion characterizes passion as a strong inclination toward a self-defining activity that an individual enjoys and in which they invest time and energy (Vallerand, 2015). The model posits two distinct pathways of internalization: Harmonious passion and Obsessive passion.

Harmonious passion results from an autonomous internalization of the activity into the individual’s identity. Exercisers engage in the activity freely, without external contingencies, leading to high positive affect, self-determined motivation, and overall well-being (Curran et al., 2015; Vallerand et al., 2003). Conversely, obsessive passion derives from a controlled internalization stemming from intrapersonal or interpersonal pressures, such as the need for social acceptance or a contingency-based self-esteem. While both types of passion are highly energizing, harmonious passion consistently facilitates superior exercise performance, enjoyment, and satisfaction, whereas obsessive passion may trigger rigid persistence accompanied by psychological distress (Curran et al., 2015; Teixeira et al., 2021b).

### 1.4 Intention as proxy for long-term adherence

To fully understand how affective responses and passion translate into actual adherence, it is essential to consider the cognitive antecedents of behavior. Grounded in behavioral models such as the Theory of Planned Behavior (Ajzen, 1991), intention is conceptualized as the most proximal and immediate predictor of actual behavior. Intention captures the motivational factors that influence a behavior, representing how hard people are willing to try and how much effort they plan to exert to perform the activity. In the context of fitness, an individual who experiences hedonic pleasure through preferred exercise intensities and develops a harmonious passion for the activity will subsequently formulate a stronger cognitive intention to continue exercising in the future. Therefore, despite the well-documented intention-behavior gap, whereby positive intentions do not always perfectly translate into actual execution, tracking the intention to continue exercising provides a useful and proximal proxy for understanding future exercise behavior.

### 1.5 Literature gap and study objectives

Despite these theoretical advancements, the intersection of these variables remains somewhat unexplored. The study of affective responses during physical exercise has gained renewed momentum (Ekkekakis and Brand, 2018), and its exploration within the Portuguese context is still in its infancy (Teixeira et al., 2021a). Furthermore, the dimensions of passion have traditionally been investigated in competitive or adapted sports contexts (Teixeira et al., 2021b) rather than among general fitness center exercisers (Rodrigues et al., 2021). To date, no study has empirically connected the preference for and tolerance of exercise intensity with harmonious and obsessive passion to predict the intention to continue exercising. To lay a foundation for understanding these relationships, this analytical cross-sectional study aims to examine the cross-sectional associations between preference for, and tolerance of exercise intensity, passion, and the intention to continue exercising among already active fitness center members. Specifically, we formulated our hypotheses to align with our analytical strategy: (a) Preference for and tolerance of exercise intensity will exhibit positive bivariate associations with both passion and the intention to continue exercising; and (b) Harmonious passion (positively) and obsessive passion (negatively) will explain the variance in the intention to continue exercising across distinct, stratified profiles of intensity preference and tolerance.

## 2 Materials and methods

### 2.1 Participants

The present study employed a cross-sectional design, collecting data at a single time point. The sample comprised 284 voluntary participants, aged between 18 and 68 years (*M* = 29.84, SD = 9.27). Regarding participant descriptors, the sample included 140 females and 144 males, based on biological sex recorded at birth. All participants were regular fitness center members, with exercise experience ranging from 1 to 30 years (*M* = 5.81, SD = 6.51). Their weekly training frequency ranged from 1 to 8 sessions (*M* = 3.81, SD = 1.51), with session durations varying between 30 and 180 min (*M* = 65.64, SD = 23.29).

The most common fitness activity reported was gym workouts (63.5%), followed by group classes (22.7%) and personal training sessions (13.8%). Regarding the perceived intensity during practice, the majority of participants reported exercising at a moderate intensity (59.2%), followed by vigorous (31.9%) and light (8.9%) intensities. Inclusion criteria for participation were: (a) being at least 18 years old; (b) exercising in a fitness center settings for a minimum of 3 months; and (c) exercising at least once a week.

### 2.2 Procedures

Prior to data collection, the study was reviewed and approved by the Ethics Committee of the Polytechnic Institute of Leiria (Approval No. 35/2021). The data collection procedure involved three main phases. In the first phase, approximately 10 fitness center managers were contacted to explain the study’s objectives and obtain authorization for the researchers to invite their members to participate. After obtaining authorization from only two clubs, the second phase of data collection began. In this phase, potential participants were informed about the nature and purpose of the study, the voluntary nature of their participation, and the guarantee of anonymity. They were also informed of their right to withdraw from the study at any time. In the third phase, questionnaires were distributed to the participants using the members’ registry. To prevent missing data, the online survey was configured with mandatory response fields, requiring participants to answer all questions in the instrument before advancing to the next section. The surveys collected sociodemographic data, exercise frequency, feelings regarding exercise practice, and perspectives on future physical activity. All ethical and deontological protocols were followed in accordance with Article 21 of Law No. 58/2019 of the Portuguese Republic Assembly. The collected data were used exclusively for research purposes and destroyed upon the completion of the study’s academic and scientific objectives.

### 2.3 Measures

Preference and Tolerance: The Portuguese version of the Preference for and Tolerance of Exercise Intensity Questionnaire (Teixeira et al., 2021a) was used to assess intensity preference (example item: “I prefer to train at a low intensity for a longer period than to train at a high intensity for a shorter period”) and tolerance (example item: “Feeling tired during exercise is my signal to slow down or stop”). This 10-item questionnaire is rated on a 5-point scale ranging from 1 (Strongly Disagree) to 5 (Strongly Agree). In the present study, the subscales demonstrated adequate internal consistency (Preference: α = 0.77; Tolerance: α = 0.79).

Passion: The Portuguese version of the Passion Scale (Rodrigues et al., 2022) was used to measure harmonious and obsessive passion. Composed of 12 items that measure harmonious passion (example item: “This activity is in harmony with other activities in my life”) and obsessive passion (example item: “I have an almost obsessive feeling toward this activity”). Participants rated each statement on a 7-point scale ranging from 1 (Strongly Disagree) to 7 (Strongly Agree). In the current sample, both subscales showed adequate internal consistency (Harmonious passion: α = 0.72; Obsessive passion: α = 0.81).

Intention: The Portuguese version validated by Rodrigues et al. (2020) was used to assess the intention to continue exercising. The questionnaire comprises three items (example item: “I intend to continue exercising for the next 6 months as I currently do, or in a very similar way”). Participants indicated their level of agreement on a 5-point scale ranging from 1 (Definitely No) to 5 (Definitely Yes). This scale demonstrated excellent internal consistency in the present study (α = 0.95).

### 2.4 Statistical analysis

Questionnaire data were exported and analyzed using IBM SPSS Statistics (version 23). Participants with missing data exceeding 5% were excluded. Measures of central tendency and dispersion, along with skewness and kurtosis values, were calculated. A univariate normal distribution was accepted since skewness and kurtosis values fell within the −2/+2 and −7/+7 ranges, respectively, as recommended by Kline (2011). To analyze the sample according to exercise intensity preference and tolerance, data were divided into two ranges for each trait score: light-to-moderate intensity (scores 5–14) and moderate-to-vigorous intensity (scores 15–25). Instead of crossing these dimensions into a 2 × 2 design, the global sample was independently stratified twice: once based on the tolerance scores and once based on the preference scores. This strategy resulted in four overlapping analytical strata (two tolerance profiles and two preference profiles), following the previous indications described by Carlier and Delevoye-Turrell (2022). This person-centered, categorical approach was deliberately chosen over continuous interaction modeling to prioritize ecological validity and practical application. By establishing distinct intensity profiles, we aimed to provide exercise professionals with recognizable phenotypic typologies that can be readily translated into tailored exercise prescriptions in real-world fitness settings. Planned Pearson’s bivariate correlations were calculated to test specific *a priori* hypotheses regarding the relationships between the study variables within each intensity trait group. Given the targeted nature of these comparisons and our focus on magnitude estimation rather than strict null-hypothesis testing, no alpha-level adjustments for multiple comparisons were applied. Instead, 95% confidence intervals (95% CI) were calculated for all correlation coefficients to transparently report the precision, uncertainty, and effect size of the associations. Statistical significance was set at *p* < 0.05. In the final step, linear regression analyses were performed, with intention as the dependent variable and harmonious and obsessive passion as independent variables. Four separate models were tested, corresponding to the four groups created based on intensity preference and tolerance. Prior to interpreting the models, preliminary analyses were conducted to ensure no violation of the assumptions of residual normality, linearity, and homoscedasticity, while the absence of influential cases and independence of residuals were confirmed by Durbin-Watson statistics ranging between 1.62 and 1.95. The explained variance (*R*^2^ and adjusted *R*^2^), unstandardized (B) and standardized (β) coefficients, 95% confidence intervals (95% CI) for B, exact *p*-values, and Variance Inflation Factors were calculated to assess the explanatory capacity of passion on exercise intentions. Statistical significance was set at *p* < 0.05.

## 3 Results

To examine the specific roles of intensity traits, the global sample (*N* = 284) was independently stratified into light-to-moderate and moderate-to-vigorous profiles for exercise tolerance (*n* = 154 and *n* = 130, respectively) and exercise preference (*n* = 87 and *n* = 197, respectively). Preliminary analysis confirmed the normal distribution of the data across all four overlapping strata, as skewness and kurtosis values fell within the acceptable cutoff ranges. As shown in Table 1, descriptive statistics revealed that harmonious passion consistently presented higher mean scores compared to obsessive passion across all intensity groups. Notably, the highest mean score for harmonious passion (*M* = 5.47, SD = 1.08) was observed in the moderate-to-vigorous preference group. Similarly, while obsessive passion also recorded its highest mean in this same moderate-to-vigorous preference group (*M* = 3.27, SD = 1.29), its overall scores remained lower and closer to the midpoint of the scale across all profiles.

**TABLE 1:** Descriptive analysis of the study variables.

Groups/variables	M	SD	S	K
**Panel A: stratification by tolerance**				
Light-to-moderate tolerance (n = 154)				
Tolerance	12.05	1.63	−0.63	−0.34
Preference	18.08	3.40	−0.34	0.09
Obsessive passion	3.20	1.29	0.21	−0.74
Harmonious passion	5.41	1.09	−0.91	1.75
Intention	4.29	0.89	−1.53	2.34
Moderate-to-vigorous tolerance (n = 130)				
Tolerance	16.79	1.66	0.72	−0.27
Preference	14.62	3.40	−0.04	−0.22
Obsessive passion	3.23	1.29	0.39	−0.80
Harmonious passion	5.46	1.09	−0.63	0.02
Intention	4.26	0.79	−1.08	0.81
**Panel B: stratification by preference**				
Light-to-moderate preference (n = 87)				
Tolerance	16.11	2.73	0.49	−0.41
Preference	12.02	1.94	−1.27	1.66
Obsessive passion	3.08	1.29	0.49	−0.57
Harmonious passion	5.37	1.12	−0.60	0.12
Intention	4.16	0.87	−1.24	1.64
Moderate-to-vigorous preference (n = 197)				
Tolerance	13.38	2.53	0.20	0.05
Preference	18.48	2.55	0.44	−0.30
Obsessive passion	3.27	1.29	0.21	−0.80
Harmonious passion	5.47	1.08	−0.86	1.40
Intention	4.33	0.82	−1.42	1.97

M, Mean; SD, standard deviation; S, Skewness; K, Kurtosis. The displayed strata overlap, as participants were independently categorized into both a tolerance group and a preference group based on their subscale scores.

Pearson’s bivariate correlations (see Table 2) were analyzed to determine the relationships between tolerance, preference, harmonious passion, obsessive passion, and intention within each group. In the light-to-moderate tolerance group, tolerance was negatively and significantly correlated with preference, while harmonious passion demonstrated a positive and significant correlation with intention. For the moderate-to-vigorous tolerance group, harmonious passion exhibited a positive correlation with intention (*r* = 0.48, 95% CI [0.34, 0.60], *p* < 0.01). Furthermore, preference maintained a significant negative correlation with tolerance across both preference groups. Its positive association with the intention to continue exercising was only statistically significant within the moderate-to-vigorous preference group (*r* = 0.14, 95% CI [0.00, 0.28], *p* < 0.05).

**TABLE 2:** Bivariate correlations between the study variables.

Groups/variables	1	2	3	4	5
**Panel A: stratification by tolerance**					
Light-to-moderate tolerance (n = 154)					
1. Tolerance	1	1	1	1	1
2. Preference	−0.35** [−0.48, −0.21]				
3. Obsessive passion	−0.04 [−0.20, 0.12]	−0.03 [−0.19, 0.13]			
4. Harmonious passion	0.01 [−0.15, 0.17]	0.04 [−0.12, 0.20]	0.24** [0.09, 0.38]		
5. Intention	−0.06 [−0.22, 0.10]	0.18* [0.02, 0.33]	0.08 [−0.08, 0.24]	0.30** [0.15, 0.44]	
Moderate-to-vigorous tolerance (n = 130)					
1. Tolerance	1	1	1	1	1
2. Preference	0.44** [−0.57, −0.29]				
3. Obsessive passion	0.04 [−0.14, 0.21]	0.13 [−0.05, 0.29]			
4. Harmonious passion	−0.12 [−0.29, 0.05]	0.14 [−0.04, 0.30]	0.28** [0.11, 0.43]		
5. Intention	−0.08 [−0.25, 0.10]	0.17 [−0.01, 0.33]	−0.03 [−0.20, 0.14]	0.48** [0.34, 0.60]	
**Panel B: stratification by preference**					
Light-to-moderate preference (n = 87)					
1. Tolerance	1	1	1	1	1
2. Preference	−0.31** [−0.49, −0.11]				
3. Obsessive passion	0.02 [−0.19, 0.23]	0.05 [−0.17, 0.26]			
4. Harmonious passion	−0.04 [−0.25, 0.17]	0.08 [−0.14, 0.28]	0.28** [0.08, 0.47]		
5. Intention	−0.03 [−0.24, 0.18]	0.15 [−0.06, 0.35]	0.06 [−0.15, 0.27]	0.28** [0.07, 0.46]	
Moderate-to-vigorous preference (n = 197)					
1. Tolerance	1	1	1	1	1
2. Preference	−0.45** [−0.55, −0.33]				
3. Obsessive passion	0.05 [−0.09, 0.19]	−0.06 [−0.20, 0.08]			
4. Harmonious passion	0.04 [−0.10, 0.18]	0.05 [−0.09, 0.19]	0.24** [0.11, 0.37]		
5. Intention	−0.01 [−0.15, 0.13]	0.14* [0.00, 0.28]	0.01 [−0.13, 0.15]	0.42** [0.30, 0.53]	

95% confidence intervals are presented in brackets.

*p < 0.05; **p < 0.01.

Following the bivariate correlation analysis, linear regression models were conducted to determine the explanatory capacity of harmonious and obsessive passion on the intention to continue exercising within each specific intensity trait profile (Table 3). Across all four groups, harmonious passion consistently demonstrated a positive and significant association with the intention to continue exercising. The models explained between 8% and 26% of the variance in intention. In the moderate-to-vigorous tolerance group, obsessive passion presented a significant negative association with intention (β = −0.18, *p* = 0.024), suggesting that highly demanding physical states coupled with obsessive regulatory pressures may negatively associated with adherence intentions.

**TABLE 3:** Linear regression models predicting intention to continue.

Model	B	95% CI	SE	β	*P*-value	VIF	*R*^2^ (Adj *R*^2^)	F (df)
Panel A: stratification by tolerance								
Light-to-moderate tolerance (*n* = 154)							0.09 (0.08)	7.47 (2,151)
Constant	2.97	[2.25, 3.68]	0.36		<0.001			
Obsessive passion	0.01	[−0.10, 0.12]	0.06	0.01	0.906	1.06		
Harmonious passion	0.24	[0.11, 0.37]	0.07	0.30	<0.001	1.06		
Moderate-to-vigorous tolerance (*n* = 130)							0.26 (0.25)	22.54 (2,127)
Constant	2.52	[1.90, 3.14]	0.31		<0.001			
Obsessive passion	−0.11	[−0.21, −0.02]	0.05	−0.18	0.024	1.09		
Harmonious passion	0.38	[0.27, 0.50]	0.06	0.53	<0.001	1.09		
Panel B: stratification by preference								
Light-to-moderate preference (*n* = 87)							0.08 (0.06)	3.54 (2,84)
Constant	3.01	[2.10, 3.93]	0.46		<0.001			
Obsessive passion	−0.01	[−0.16, 0.13]	0.07	−0.02	0.848	1.09		
Harmonious passion	0.22	[0.05, 0.39]	0.09	0.28	0.011	1.09		
Moderate-to-vigorous preference (*n* = 197)							0.19 (0.18)	22.28 (2,194)
Constant	2.68	[2.12, 3.24]	0.29		<0.001			
Obsessive passion	−0.06	[−0.15, 0.02]	0.04	−0.10	0.141	1.06		
Harmonious passion	0.34	[0.24, 0.44]	0.05	0.45	<0.001	1.06		

B, unstandardized coefficient; 95% CI, 95% confidence interval for B; SE, standard error; β, standardized coefficient; VIF, Variance Inflation Factor; *R*^2^, R-squared; Adj *R*^2^, Adjusted R-squared; F, F-statistic; df, degrees of freedom. Exact *p*-values are reported.

## 4 Discussion

The primary objective of the present study was to examine the role of preference for, and tolerance of, exercise intensity in harmonious and obsessive passion, as well as the intention to continue exercising among fitness center members. By dividing the sample into distinct profiles based on intensity traits, this study provided a nuanced analysis of the cognitive and affective determinants of exercise adherence. Overall, the formulated hypotheses were partially supported, revealing theoretical distinctions between preference and tolerance, as well as between the two dimensions of passion. Regarding our second hypothesis (b), we anticipated that preference and tolerance would be positively associated with both harmonious and obsessive passion. However, our findings only partially supported this premise. As demonstrated in Table 2, while harmonious passion showed a significant positive relationship with preference in specific profiles (e.g., such as the moderate-to-vigorous tolerance group), tolerance itself did not exhibit consistent correlations with either dimension of passion. This suggests that the mere capacity to endure physical discomfort (i.e., tolerance) is not inherently linked to how one internalizes the exercise activity (i.e., passion).

In line with our third hypothesis, harmonious passion emerged as a consistent, positive, and significant correlate of the intention to continue exercising across all intensity and tolerance groups. These results corroborate recent literature, including findings by Teixeira et al. (2022), which demonstrated that harmonious passion is associated with intention and habit formation. According to the Dualistic Model of Passion (Vallerand, 2015), when an individual autonomously internalizes exercise into their identity, the activity generates high levels of positive affect, satisfaction, and enjoyment (Curran et al., 2015). Consequently, this positive affective state is cross-sectionally linked to a stronger behavioral intention to return to the fitness center setting, though longitudinal data are needed to confirm if this translates into actual adherence. Conversely, our hypothesis regarding obsessive passion was not fully supported. Instead of positively influencing intention, obsessive passion exhibited a neutral or significantly negative association with the intention to continue exercising, particularly in the moderate-to-vigorous tolerance group. This negative trajectory aligns with the conceptualization of obsessive passion as a controlled internalization driven by intrapersonal pressures (e.g., contingency-based self-esteem) (Vallerand et al., 2003). While obsessive passion may initially energize behavior, the rigid persistence and potential psychological distress associated with it, especially when combined with highly demanding physical tolerance limits, appear to compromise long-term motivational stability and behavioral intention. Perhaps the most revealing finding of this study is the functional divergence between intensity preference and tolerance. Our first hypothesis posited that both traits would be positively associated with intention; however, the data only partially supported this. While preference for exercise intensity showed a positive and significant association with intention (specifically in the light-to-moderate tolerance and moderate-to-vigorous preference groups), tolerance exhibited no statistically significant relationship with the intention to continue exercising. This distinction is critical and can be interpreted through Hedonic Theory (Ekkekakis and Dafermos, 2012). Preference reflects the intensity an individual chooses to maximize pleasure. When exercise aligns with these preferred levels, the resulting positive affective valence acts as a powerful correlate of future behavioral intention. In contrast, tolerance represents the capacity to endure physical stress. Our results suggest that the sheer ability to withstand high-intensity discomfort (tolerance) does not translate into a cognitive desire to repeat the behavior. While recognizing that intentions do not invariably lead to action, our findings tentatively suggest that prescribing exercise based solely on an exerciser’s maximum physical capacity, without considering their hedonic preference, may be suboptimal for fostering long-term exercise intentions.

### 4.1 Limitations of the present study

Despite the theoretical contributions of this study, several methodological limitations must be acknowledged. First, the cross-sectional design fundamentally precludes causal inferences or directionality; reverse causality remains plausible, as individuals with stronger exercise intentions may naturally perceive their involvement as more harmonious, or both constructs might simply reflect a general positive self-concept. Furthermore, by treating psychological constructs as stable, between-person characteristics, this study overlooks the highly dynamic nature of exercise behavior. Ecological momentary assessment research indicates that 50%–75% of the variance in intentions occurs within persons, fluctuating across days, situations, and specific exercise episodes (Conroy et al., 2013; Dunton, 2017). Our static measurements fail to capture this intra-individual variance. This issue is compounded by our analytic strategy, which categorized continuous preference and tolerance scores into discrete intensity groups. This approach inherently reduces statistical information and creates artificial boundaries; future investigations should retain these variables as continuous predictors and utilize interaction terms to test their joint roles more accurately. Second, actual exercise adherence, dropout prevention, or long-term behavioral persistence were not directly monitored. The dependent variable was limited to a broad, distal intention to continue exercising over the next 6 months, lacking behavioral specificity regarding activity type, frequency, intensity, or contextual barriers. This renders the outcome closer to a global motivational self-report than a behavioral criterion. Similarly, our analyses aggregated all fitness center members without differentiating between specific activities (e.g., resistance training vs. group classes). Because intensity preferences and affective responses are highly activity-specific, this global score masks critical contextual nuances. Crucially, the core mechanism implied by the hedonic perspective remains unobserved. The study lacks momentary assessments of pleasure, displeasure, affective valence, or perceived exertion captured before, during, or after exercise. Inferring hedonic regulation strictly from trait-like scores fails to capture situation-specific processes, highlighting the need for future repeated-measures protocols to track intra-exercise affect in real time. Finally, a significant selection bias limits the generalizability of our findings. Participants were exclusively recruited from regular fitness center members with at least 3 months of experience, essentially describing a “survivor” cohort. Consequently, these data do not represent the psychological barriers faced by sedentary populations or early dropouts. Lastly, the prominent relationship between harmonious passion and intention should be interpreted with caution. Because both constructs represent positive, self-referential motivational evaluations of the same activity, their strong statistical association may partially reflect conceptual overlap or shared method variance rather than a longitudinal process of exercise maintenance.

## 5 Conclusion

This study demonstrates that intensity preference and harmonious passion are cross-sectionally associated with a general intention to continue exercising among individuals who are already active in a fitness center setting. However, given the structural and cross-sectional nature of this design, these findings do not provide evidence of actual exercise adherence over time, within-person fluctuations, or the conversion of intention into behavior. Consequently, any practical recommendations derived from this study must remain strictly preliminary rather than intervention- or evidence-based. Exercise professionals should interpret these associations cautiously. While tailoring workout environments to individual intensity preferences and fostering harmonious passion are plausible goals, longitudinal and experimental validation is strictly required before these concepts can be translated into definitive clinical or practical exercise prescription guidelines.

## Data Availability

The data presented in this study are available on request from the corresponding author, provided there is a clear and justified research objective. The data are not publicly available due to privacy and ethical restrictions regarding participant anonymity.
